# Researches and Simulation of Elastic Recovery Phenomena during Roller Burnishing Process of Macro-Asperities of Surface

**DOI:** 10.3390/ma13225276

**Published:** 2020-11-21

**Authors:** Agnieszka Kułakowska, Łukasz Bohdal

**Affiliations:** Department of Mechanical Engineering, Koszalin University of Technology, Racławicka 15-17 Street, 75-620 Koszalin, Poland; lukasz.bohdal@tu.koszalin.pl

**Keywords:** numerical analysis, roller burnishing process, roughness, surface state, previous treatment, outline deviations, elastic recovery

## Abstract

The paper presents preliminary studies of a new innovative surface treatment method—the process of roller burnishing of macro-irregularities of the surface. As part of the work, the possibility of plastic shaping of the surface macrostructure with indentations (plateau structure), which will show anti-wear properties through appropriate surface shaping and the compressive stress state in the product’s top layer, was investigated. The essence of the paper is the analysis of one of the aspects of the application of this processing method, i.e., the influence of the elastic recovery of the product on its technological quality measured by dimensional deviation. The main objective of the work is to develop adequate methods and mathematical models to enable the design of the macro-asperities of the surface burnishing process to maintain the dimensional tolerance of the shaped parts. The results of dependencies of elastic recovery of the asperities and the deviation of height, Δ*h*_t_, for sample depths of burnishing were presented. The model tests of the elastic recovery of the model material using the visioplasticity method show that with the increase of the value of the vertical surface asperities, the value of the elastic recovery of the material decreases. The increase of the deviation of the asperities’ height causes a decrease in the value of elastic recovery. With the increase of the value of the vertical angle of the surface roughness, the value of the elastic recovery of the material is smaller.

## 1. Introduction

Roller burnishing is used increasingly as a finishing operation [1,2,3,4,5,6]. It is known that the surface state after previous treatment has significant influence on its quality after roller burnishing [7,8].

The effect of burnishing is to reduce the roughness of the profile while hardening the surface layer of the product and the formation of compressive stress in it [1,2,3,9]. In the process of burnishing several interacting processes occur simultaneously, phenomena present in the material deformation areas affected on the conditions in the contact zone, and those on some of the burnishing conditions, which also affects its complexity [5,10].

Review of the literature shows that work on the burnishing process has been conducted by many researchers. Several works have investigated the effect of the burnishing process to improve the properties of the parts’ mechanical properties, e.g., increased hardness [2], higher wear resistance [6], surface quality [9,11], and increased maximum residual stress in compression. The parameters affecting the surface finish are burnishing force, feed rate, ball material, number of passes, workpiece material, and lubrication [6]. References [1,2,3,4] showed that the burnishing force and the number of tool passes are the most predominant of the parameters that have an effect on the surface roughness of the workpiece during the burnishing process.

Determining roller burnishing parameters also causes difficulty in achieving the required quality, because the measurement of process parameters (determining the technological quality such as temperature field, stress field, structural changes, the change in the dimensions under the influence of elastic deformation, etc.) during the burnishing process in the current state technique is impossible.

The process parameters can only be inferred from the properties of the product after the process. As a result of burnishing, there are dimensional changes caused by the following factors:

1. Plastic deformation and the decrease in surface roughness,
2. The concentration and structure squeeze,
3. An axial elongation of the product due to plastic deformation and residual stress generated in the surface layer [6],
4. Moving the material after moving away the burnishing element and increasing the asperities height.

Despite numerous works on burnishing treatment, the problem of springback of the material after unloading (or moving away) the burnishing tool, has been considered in a few studies [12,13]. The problem of elastic recovery is most often occupied by researchers working on pressing an indenter into the material [14,15,16,17,18,19], and less frequently, rolling [20] or the process of sheet metal forming [21] or cutting [22].

Stilwel and Tabor [18] found that when a spherical indenter is pressed into a plastic material, it creates a cavity, then after it is pulled back, there is an elastic return of the material of both the indenter and the cavity. Shape changes can be described in relation to the Hertz equation for the elastic deformation of a spherical surface. Chodór, in his work [22], using the results from numerical analyses, shows the elastic recovery of the material during the process of micro-cutting and sliding burnishing. Skalski [23] noticed that during the propagation of the plastic area (in the case of pressing a rigid cylinder into a body with a flat surface), a narrow elastic area appears under the cylinder on a plastic substrate. This area does not plasticize even during increasing load.

Despite the large number of works on burnishing, elastic recovery has not been considered. There are works concerning elastic recovery during the scratch test [14,24] but not during roller burnishing. Also, in many papers, modeling and numerical analysis of the process were presented [25,26,27,28,29,30,31] but without considering the outline of the surface after previous treatment and material elastic recovery. Reference [32] describe the effects of ball-end hard milling to a variation of the lubrication situation. At the reversal, roller burnishing process parameters on the surface point to micro-contacts at the maximum rolling speed elasto-roughness of the raceways of constant velocity joint shafts. The authors of Reference [33] developed a three-dimensional (3D) numerical finite element model of the ball burnishing process including real surface integrity descriptors resulting from a ball-end-milled AISI 1038 surface in the target workpiece. Specifically, its periodical topological features were used to generate the surface geometry. Different models varying the effect of the coefficient of friction and the direction of application of burnishing passes with regards to the original milling direction were calculated. Reference [34] reports the results of experimental studies on the impact of ball burnishing parameters on the roughness, microstructure, and microhardness of the surface layer of laser-cut C45 steel parts. The distribution of residual stresses generated in the surface layer of these parts were analyzed. The superficial effect of hardening caused after vibration-assisted ball burnishing and its consequences in the tensile behavior of a carbon steel material were studied in Reference [35]. Experiments using different amplitudes and new forces were encouraged to obtain more information about how the material can be modified optimally through vibration-assisted ball burnishing.

Burnishing of the macro-irregularities of a surface is a variation of the typical burnishing process, however it differs in the scope of influence on the surface layer of the product. The research on this new process was undertaken due to the conducted preliminary research, which gave very favorable operational effects of the surface layer shaped in this way. It can be seen that the depth of strengthening the surface layer can be even a few mm, while for a typical burnishing process, only a part of a mm or a few µm. Additionally, the literature shows how to solve the problem of the kinematics of the roughness of the material for the triangular asperities of the surface crushed (burnished) with a flat punch under various friction conditions [36,37,38]. Kukiełka [10,39,40] stated that the kinematics of the smoothing of the asperities depends only on the apex angle of the rough surface and determined the angle boundaries for which there is a change in the flow mechanism.

**Figure 1:**
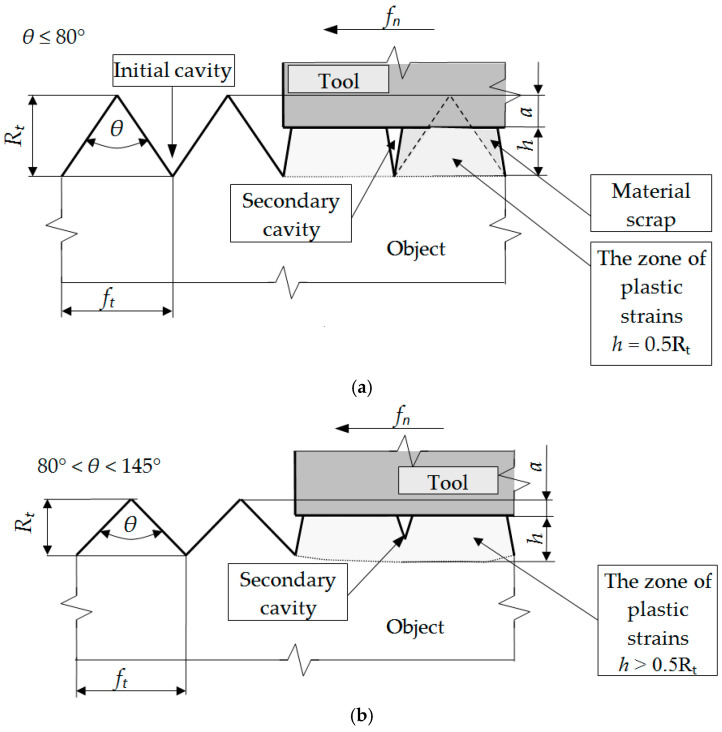

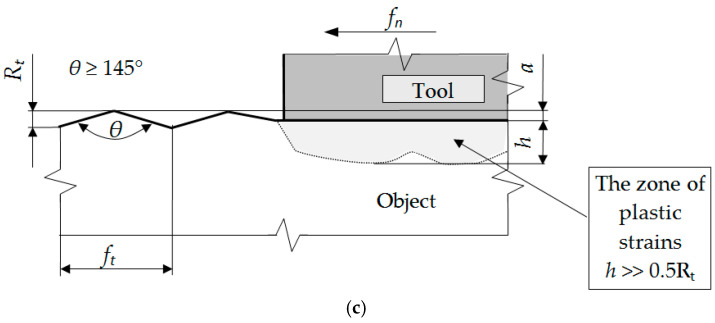
The influence of the vertical angle of a symmetrical triangle asperity on the depth of strain zone and surface roughness profile after burnishing: *θ* ≤ 80° (**a**), 80° < *θ* < 145° (**b**), *θ* ≥ 145° (**c**) [39].

The author of Reference [39] qualitatively distinguished three different cases of flow of the surface layer of material. The considerations were carried out with the assumption that the surface asperity after the previous treatments is triangular, symmetrical, and regular. The research shows that in the burnishing process, these cases depend only on the vertical angle *θ* of the asperity (Figure 1):

- For vertical angles *θ* ≤ 80°, the material core remains undistorted. The indentations of the asperity do not rise, and the deformation of the material occurs only within the unevenness. A gap (discontinuity planes) with the depth 0.5 Rt that separates deep inequalities can be seen. As a result of the material flowing sideways, the surface flattens out.
- For the vertical angles 80° < *θ* < 145°, the zone of plastic deformation includes the material core. Gaps are still visible at the junction of adjacent burrs but of less depth. The asperity valleys rise and total strain in the contact zone of neighboring material overflows.
- For angles *θ* ≥ 145°, as a result of deformation of asperity and the material core, the surface is evened out. This is not at the expense of the material outflows towards the sides of the asperity. There are no planes of material discontinuity in the surface layer. The rise of the indentation is equal to the decrease in the top of the asperity.

A significant problem is to ensure the regularity of the outline and the appropriate vertical angle *θ* of the shaped irregularities in the treatment preceding burnishing. Due to the type of the machined part and its intended use, the value of the feed, *f_t_*, in the preceding machining and the value of the vertical angle *θ* of the asperity should be selected each time.

In previous paper [12] on the initial stages of the experiment related to elastic recovery issues, we analyzed the influence of the deviations of the height and distance of the asperities after turning on the deviation of the height of the asperities of the surface as well as elastic recovery after burnishing. Research was performed for different burnishing depths. The calculations were performed for apex angles *θ* = 90° and *θ* = 120°. The received results showed that the elastic recovery increases as the burnishing depth increases. Each of the asperities were analyzed separately. The analysis of the state of knowledge shows that none of the researchers analyzed the problem of elastic recovery, which is a novelty and originality of the presented article. In the paper, FEM (Finite Element Method) analysis with using the dynamic explicit method for the roller burnishing process was established. The surface state after turning (as previous treatment) was taken into account. The results of dependencies of elastic recovery of the asperities and the deviation of height, Δ*h_t_*, for exemplary burnishing depth is presented. The influence of the surface after previous treatment on the burnished product quality can be analyzed by developed numerical algorithms. It allows for better understanding of the phenomena which occur in the tool–workpiece contact zones. The basis for the development of guidelines for the selection of the conditions of rolling and burnishing processes considering the required technological quality of the product can be formulated. This paper consists of numerical simulations and analysis, model test, and the research on the steel C45, with the aim to understand elastic recovery as well as analysis of elastic recovery of the asperities with different vertical angles.

## 2. Idea of Elastic Recovery

**Figure 2:**
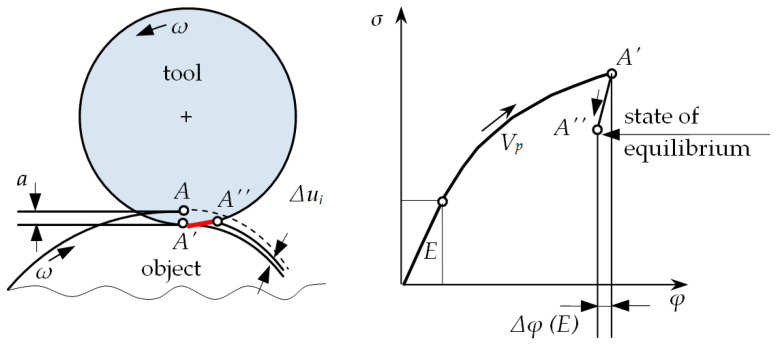
Elastic recovery during the roller burnishing process.

It is assumed that during the burnishing process, deformations of the material occur in terms of elastic and visco-plastic strains [41]. It is known that visco-plastic strains will remain in the material, while elastic strains change [41,42,43]. After the burnishing tool has passed, the phenomenon of elastic recovery occurs and visco-plastic deformations remain in the processed material, while the elastic deformations change and settle on the level corresponding to the new state of equilibrium of the object. Thus, the states of displacements, stresses, and strains in the tested object during and after the end of the process differ significantly, because after the process is completed, the nodes are displaced in the elastic range. The value of elastic deformations will decrease by the value Δ*ϕ^(E)^*, while the elastic deformations will remain at the value *ϕ^(E)^* in the surface layer of the product. This is shown in Figure 2.

During the process, the burnishing tool is plunged to the depth *a*. It can be noticed that the contact point of the tool and the workpiece material *A*, as a result of this depression, moves to the point *A′*. Then, after the rotation of the workpiece and the burnishing tool, the load is unloaded and point *A′* is moved to position *A″*. The radial distance between points *A′* and *A″* is marked as Δ*u_i_* and represents the elastic recovery of the material after burnishing, and *φ* is the actual deformation of the material.

**Figure 3:**
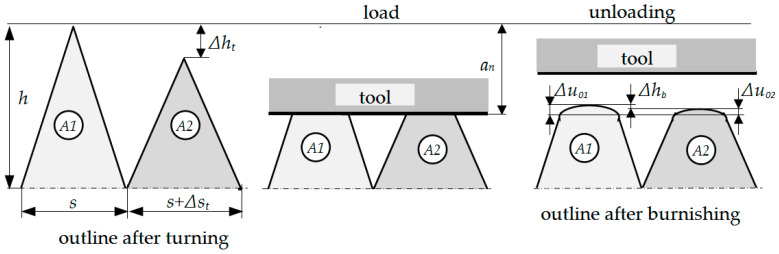
Schematic diagram of the elastic recovery of the material after burnishing: *A1*—first asperity, *A2*—second asperity after previous machining (turning), *h*—asperity’s height, Δ*h_t_*—height deviation of asperity after turning, Δ*h_b_*—asperity’s height deviation after burnishing, *a_b_*—burnishing depth, Δ*u*_01_—elastic recovery of the material of first asperity *A1*, Δ*u*_02_—elastic recovery of the material of second asperity *A2*.

The process of roller burnishing is treated in this study as a process with unknown boundary conditions in the tool–workpiece contact area. It is a geometrical and physical boundary and initial value problem. During experimental research and simulations of the process, special attention was drawn to the elastic strain of the material, which takes place after moving away the burnishing element, called elastic recovery, Δ*u_i_*. The state of the surface layer after previous treatment and after roller burnishing are considered together. They take into account two asperities, *A1* and *A2*, after the turning process, which are burnished. Asperity *A1* after turning was characterized by constant height (*h*) and distance (*s*). Asperity *A2* possesses different values of the height or distance. The difference between the asperities was called height deviation after turning (Δ*h_t_*) and between distances: distance deviation after turning (Δ*s_t_*). Similarly, deviations were defined after burnishing and marked as Δ*h_b_* and Δ*s_b_*. The schematic diagram of elastic recovery in joint analysis of the surface after turning and burnishing is presented in Figure 3.

## 3. Researches of the Influence of Height and Distance Deviations on the Elastic Recovery

### 3.1. Experimental Researches—Materials and Methods, Discussion

The experiment was conducted for the following input factors: height deviation (Δ*h_t_*) and distance deviation (Δ*s_t_*) of the surface asperities after rolling, determined in compliance with Table 1, and the vertical angle of asperities was constant, *θ* = 60°. The five-level rotatable experiment plan was used to set up the values of the deviations.

**Figure 4:**
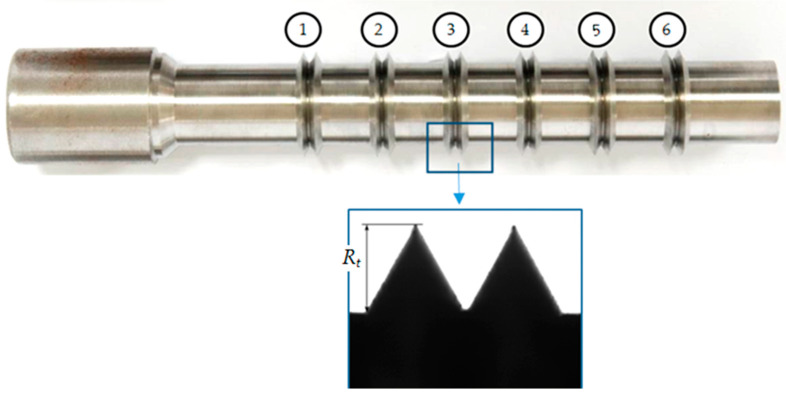
General view of the shaft with the samples after turning process and zoom of the sample (nb 3) after turning process, where *R_t_* is the asperity height.

**Figure 5:**
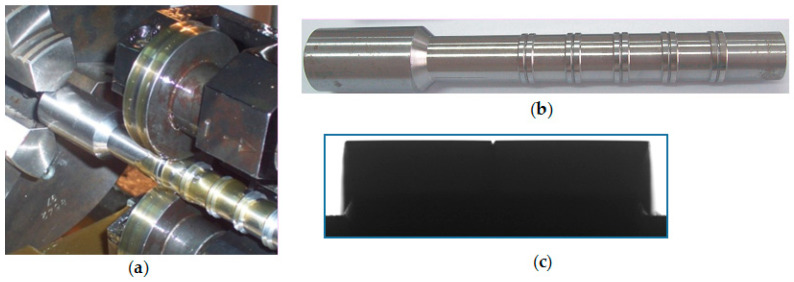
Burnishing position (**a**), general view of the shaft with the samples after burnishing (**b**), zoom of the asperities after roller burnishing (**c**).

The values of the input factors were changed in the following ranges: Δ*h_t_* = 0.01–0.04 mm, Δ*s_t_* = 0.05–0.2 mm. The tests were repeated three times for each point of the experiment plan. Tests were performed at room temperature (T = 19 °C). The external surfaces of the samples made of steel C45 were prepared in the turning process on a NEF 520 numerical lathe in “TEPRO” Vacuous Technology Plant in Koszalin, with a VCMT 160402 machining plate. The following machining parameters were used: velocity *v_t_* = 200 m/min and feed *f_t_* = 0.15 mm/rev in rough machining and *v_t_* = 200 m/min, *f_t_* = 0.1 mm/rev in finishing. Six samples were made on the shaft (Figure 4). Each sample included two asperities, which differed in the value of the height and distance deviations, according to the experiment plan. The accuracy of the shaping of the outline was verified on a Werth optoelectronic microscope. Figure 5 shows an example of a shaft with samples prepared in accordance with the experiment plan, Table 1 (first 6 implementations). The values of the height and distance deviations obtained in measurements were not significantly different (at the significance level *α* = 0.05) from those planned on the basis of single factor analysis of variance.

Next, the samples were burnished using a Fette T27 tangent burnishing head with high stiffness to reduce the influence of dimensional changes of the head on results. The burnishing depth for each sample was calculated separately, while assuming that it is *a* = 0.5 *R_t_*, where *R_t_* is the asperity height without a deviation (constant one).

**Figure 6:**
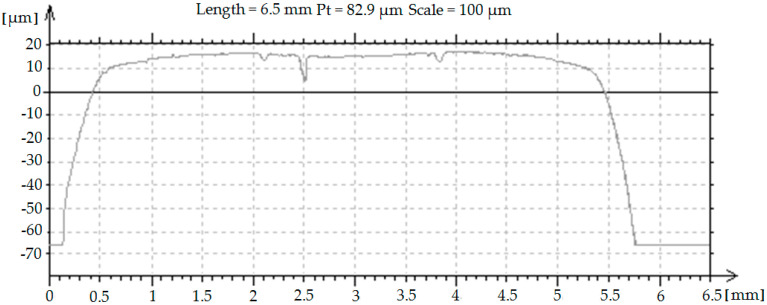
Exemplary profile of the asperities after roller burnishing.

After the burnishing process, the values of parameters Δ*u*_0_ were calculated and profiles with profilometer T8000 were obtained. Exemplary profile of burnished asperities is presented in Figure 6.

The results of the measurements were developed statistically in the Experiment Planner program developed by Kukiełka L. and Kukiełka S. [44,45,46,47,48,49,50]. The following regression equation is obtained of the dependence of the material elastic recovery after burnishing (Δ*u*_0_) from the deviations of height (Δ*h_t_*) and distance (Δ*s_t_*) of asperities after turning, for correlation coefficient *R* = 0.985:

(1) $$\begin{array}{l} \Delta u_{0}=0.0007\Delta s_{t}^{}-0.013\Delta h_{t}-2.423\times 10^{-14}\Delta s_{t}\Delta h_{t}+\\ +0.0048\Delta s_{t}{}^{2}+0.12\Delta h_{t}{}^{2} \end{array}$$

**Figure 7:**
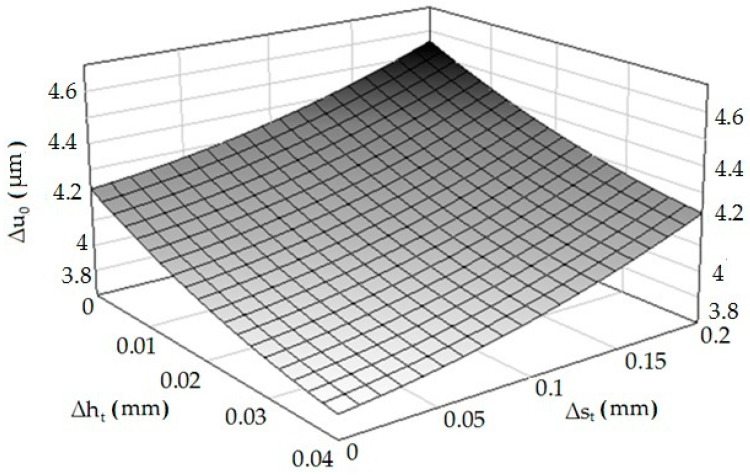
Dependence of the material elastic recovery after burnishing (Δ*u*_0_) from the deviations of height (Δ*h_t_*) and distance (Δ*s_t_*).

In order to determine the form of Equation (1), appropriate analytical and experimental tests were carried out, in the following stages: determination of the sets of tested, constants, disturbing and resulting factors, determining the range of variability (research area) of the examined factors, adoption of the class of the mathematical model of the research object, coding of researched factors, implementation of the actual research, experiment plan, the results of the experiment, elimination of results with a gross error, calculating the inter-row variance, checking the homogeneity of variance in the sample, calculating the coefficients as a function of the regression, statistical analysis of the regression function, study of the significance of the multidimensional correlation coefficient, checking the adequacy of the mathematical model, and decoding the regression function. The detailed procedure is given in References [39,41,44]. The graph of the function according to Equation (1) with the correlation coefficient *R* = 0.86 is presented in Figure 7.

It is visible that an increase of the deviation of the asperities’ height causes a decrease in the value of elastic recovery, while the opposite situation occurs when the deviation of the distance is analyzed. Knowledge about influence of determined profile parameters on the physical phenomena that occur in the burnishing process is unknown. It is important because they impact on the quality of the formed product’s surface layer, tribological properties, and fatigue issues. Many physical phenomena whose observation or measurement is very difficult or impossible take place during processes of burnishing, such as the pressure in contact zones, the friction forces, strain and stresses, elastic recovery, the slip and contact zones, and states of displacement. After passing the burnishing tool, visco-plastic deformations remain in the workpiece, while the elastic deformations change and settle at the level corresponding to the new equilibrium state of the object. Thus, the states of displacements, stresses, and strains in the tested object during and after the end of the process differ significantly, because after the process is completed, the nodes are displaced in the elastic range. In the case of higher asperities, the displacement is greater than in the case of those with a height deviation. The opposite is the case for a distance deviation. These issues are very complex and require further analysis.

### 3.2. FEM Modeling and Numerical Results

The computer models (2D and 3D) of the process in the real scale were characterized by the possibility of changing the value of the height deviation (Δ*h_t_*) and the distance deviation (Δ*s_t_*). Exemplary calculations were performed for the apex angle *θ* = 60° and the asperities feed *f* = 2.7 mm, and the deviations were changed according to Table 1. Numerical analyses were conducted with the aid of Ansys Structural, LS-Dyna and LS-Prepost programs. In the analyzed case, the object was treated as an elastic body (in terms of reversible deformations) and visco-plastic (in terms of irreversible deformations), while the tool (roller) was treated as a perfectly rigid body. Based on the results obtained, various analyses can be carried out. In the discussed case, only some of the results were presented in order to determine the impact of the burnishing process of the rough surface (with regular triangular asperities) on the value of elastic material return after the burnishing process. Moreover, in incremental material models, it was assumed that the object was made of C45 steel, for which the cumulative value of the yield stress in 2D models is described by the regression equation according to Equation (2):

(2) $$\sigma_{p}=976.95\;{\left(0.0667+\varphi_{i}^{\left(\mathrm{VP}\right)}\right)}^{0.2052}{\left[2.33\times 10^{-6}{\left({\dot{\varphi }}_{i}^{\left(\mathrm{VP}\right)}\right)}^{2}+8.83\times 10^{-5}{\dot{\varphi }}_{i}^{\left(\mathrm{VP}\right)}+1\right]}^{1.337}$$

where: yield stress is *σ_p_*, intensity of the real strain is *φ_i_*, and strain rate is ${\dot{\varphi }}_{i}$.

**Figure 8:**
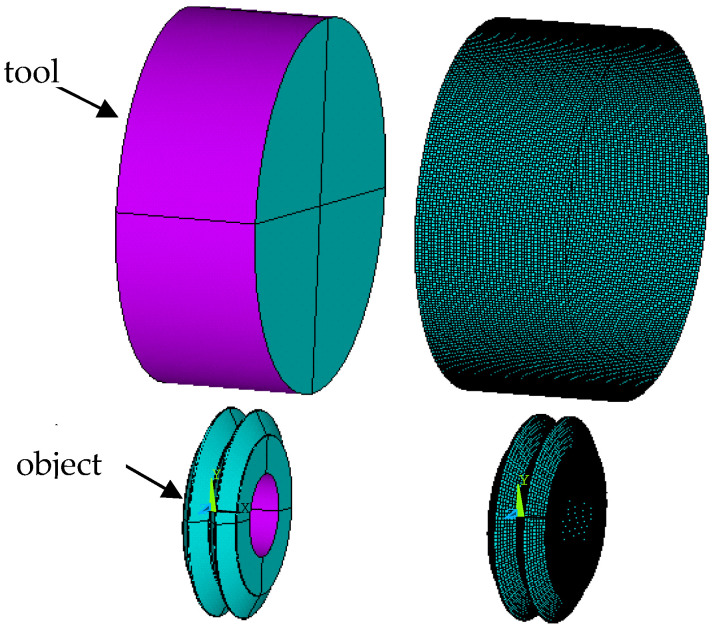
Geometry of the object, tool, and finite element mesh.

The necessary parameters for this material are Young’s modulus *E* = 210 GPa, Poisson ratio *ʋ* = 0.29, yield stress *R_e_* = 425 MPa, and hardening modulus *E_T_* = 1024 MPa. In 3D models, object discretization was performed using finite elements of the object with a linear shape function, shell elements (tool), and contact elements (contact of the tool with the object). The nonlinear, anisotropic Coulomb friction model and the coefficient of static friction *μ_s_* = 0.1 and dynamic friction *μ_d_* = 0.05 were adopted. Then, adequate boundary conditions were set—mainly concerning the displacements: tool cavity (up to the value of 0.5 of the roughness height) and the degrees of freedom for the workpiece, as well as the set rotational speed ensuring the required burnishing speed *v_b_* = 0.52 m/s. In this case, the shaft with surface asperities was rotated at *n* = 477 rpm, then the burnishing element (roll) was moved to the burnished surface. The contact of the roller with the machined surface caused the tool to rotate. In the next step, methods of solving the discrete equation of motion were introduced. For this purpose, in accordance with the adopted algorithm of explicit solution, at each analysis step, an approximation of the column vector of acceleration and velocity increment as a function of the column vector of displacement increment was introduced, all with the following dimension integration and were given the required number of steps, as well as the total calculation time *t* = 3.5 s. The geometrical model and mesh grid are shown in Figure 8. The 3D FEM model was developed according to Hill plasticity condition and low strain rates. Thermal strains for this model were approximately 2%.

The temporary yield stress in the 3D model was described with the aid of the Cowper–Symonds model. A Cowper–Symonds model allows for linear isotropic (*β* = 1), kinematic (*β* = 0), or mixed (used in this study) (0 < *β* < 1) plastic strain hardening, and the effect of the plastic strain velocity is given by the following power relation:

(3) $$\sigma_{p}={\left[1+\left({\dot{\varphi }}_{i}^{\left(p\right)}/C\right)\right]}^{m}\;\left(R_{e}+\beta E_{p}\varphi_{i}^{\left(p\right)}\right)$$

where *β* is the plastic strain hardening parameter, $R_{e}$ (MPa) is the initial static yield point, ${\dot{\varphi }}_{i}^{\left(p\right)}$ (s^-1^) is the plastic strain velocity, C (s^−1^) is the material parameter defining the effects of the plastic strain velocity, m = 1/P is the material constant defining the sensitivity of the material to the plastic strain velocity, $\varphi_{i}^{\left(p\right)}$ is the plastic strain intensity, and $E_{P}=\frac{E_{T}E}{E-E_{T}}$ is a material parameter dependent on both the plastic strain hardening modulus, $E_{T}=\partial \sigma_{p}/\partial \varphi_{i}^{\left(p\right)}$, and Young’s modulus, *E*.

**Figure 9:**
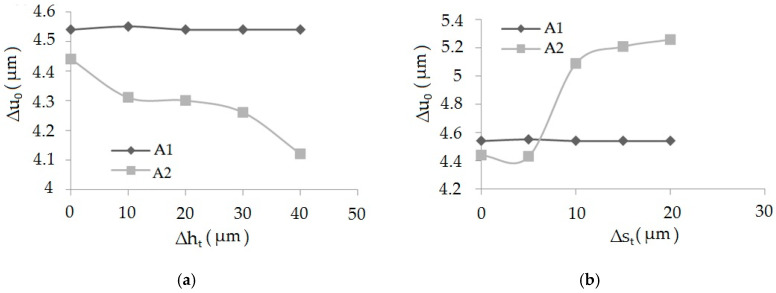
The graphs of dependencies of elastic recovery Δ*u*_1_ and Δ*u*_2_ accordingly in the asperities *A1* and *A2*, from height deviation, Δ*h_t_* (**a**), and dependencies of elastic recovery Δ*u*_1_ and Δ*u*_2_ accordingly in the asperity *A1* and *A2* from distance deviation, Δ*s_t_* (**b**).

To find the value of material elastic recovery in performed computer simulations, one had to select areas when the tool affected the subject, and after it moved away. Two kinds of analysis were performed. The first, when Δ*h_t_* (height deviation) values after the turning process were Δ*h_t_* = 0–0.04 mm and Δ*s_t_* (distance deviation) values after the turning process were Δ*s_t_* = 0, and the second, when Δ*s_t_* = 0–0.2 mm but Δ*h_t_* = 0. On the basis of computer simulations, the graphs of dependencies of elastic recovery of the first Δ*u*_1_ and the second Δ*u*_2_ asperity and the deviation of height, Δ*h_t_*, for the sample after burnishing were elaborated. The graph of dependencies of elastic recovery Δ*u*_1_ and Δ*u*_2_ in the asperities *A1* and *A2* from distance deviation Δ*s_t_* is presented in Figure 9.

It has been observed that together with the increase of the deviation of surface asperities’ height, the elastic recovery decreases. Comparing the values of elastic recovery for an asperity with constant geometry *A1* and changeable geometry *A2*, it can be seen that the value of elastic recovery was higher for asperity *A1*.

**Figure 10:**
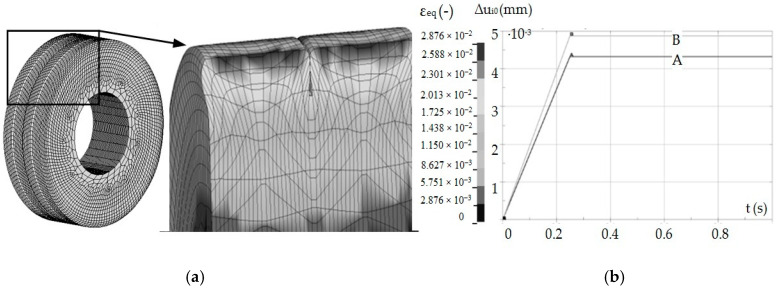
Geometric model with a mesh of finite elements and mesh deformation (**a**), elastic return of selected inequality nodes with variable geometry—A and constant—B (**b**).

Computer simulations were also performed to validate the experimental studies according to the experiment plan presented in Table 1. An example of the results obtained from the simulation is shown in Figure 10, including a geometric model with a mesh of finite elements (a), mesh deformation (b), elastic recovery of selected asperity nodes with variable geometry (A) and constant (B).

On the basis of the obtained results, an Equation (4) was statistically developed, describing the influence of height deviation and distance deviation on the elastic recovery of the material. Multidimensional correlation coefficient *R* = 0.8332.

(4) $$\Delta {\hat{\bar{u}}}_{0}=0.0048-0.0032\Delta s_{t}-0.026\Delta h_{t}-3.526\times 10^{-14}\Delta s_{t}\Delta h_{t}+0.024\Delta s_{t}^{2}+0.358\Delta h_{t}^{2}$$

**Figure 11:**
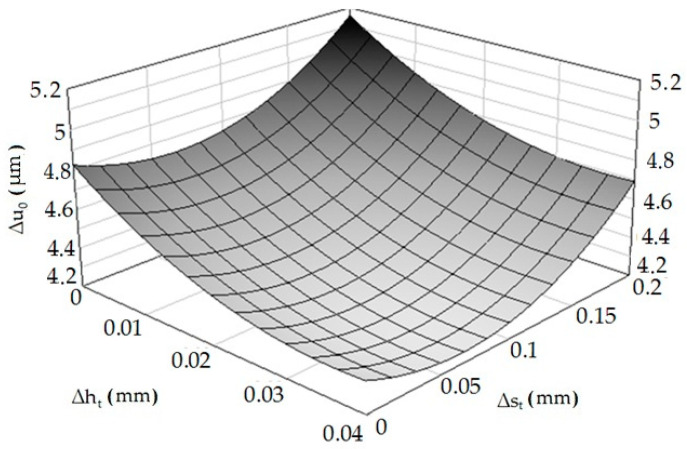
Relation of elastic material recovery after burnishing—Δ*u*_0_ = *f*(Δ*h_t_*, Δ*s_t_*).

As in the case of experimental studies, it was found that an increase of the asperities’ height deviation causes a decrease in the value of elastic recovery, while the opposite situation occurs when the distance deviation is analyzed. The results of experimental studies and computer simulations differ slightly from each other. This may result from simplifications and assumptions made when developing the computer model (e.g., refinement of the finite element mesh, shape of elements). Based on Equation (4), a spatial diagram (Figure 11) was prepared.

## 4. Researches of the Phenomenon of Elastic Recovery Depending on the Vertical Angle of the Asperities

### 4.1. Modelling Researches of the Phenomenon of Elastic Recovery 

Experimental studies with the use of a model material were carried out in order to assess the effect of the flow blocking phenomenon through adjacent irregularities on the elastic return of the surface after burnishing. The tests were carried out in accordance with the rheological, geometric, and time scales [4,36,44]. Modeling consists in replacing a real research object with a model object. The real and model objects form a system of equivalent objects whose similarity is determined by mathematical relationships. Then, the modeling results can be translated into a real research object. Physical modeling on substitute material is used in experimental qualitative and quantitative analysis. The most frequently used model material replacing metal is a non-metallic material, such as plasticine, wax, putty, etc. These are materials with much lower plastic flow resistance. Physical modeling on plasticine is used for experimental qualitative and quantitative analysis of the plastic working processes. Qualitative analysis mainly concerns the kinematics of the material flow process, while the physical analysis covers the study of force and energy parameters (force, unit pressure on the contact surface of the tool—model material). In recent years, the technique of visualizing the flow trajectory of material particles of the shaped material, the so-called method of visioplasticity, has been used. It is based on the study of plastic flow kinematics based on the observation and measurements of the coordination grid. Performing experimental tests of visualization on real metal materials is laborious and costly and requires a station with very high-pressure forces. Therefore, often in experiments, the real material is replaced by the so-called model material or numerical analyses are performed [39,42,45]. In order to ensure the condition of rheological similarity, the characteristics for the model material—plasticine—were determined. This is due to its availability, low price, easy workability, plasticity (plasticizing stresses are 100–1000 times lower than the appropriate metal stresses), the possibility of its modification and regeneration, and therefore, multiple use. The material model was developed based on the static compression test. The results of this test are approximate data due to the presence of disturbances in the measurement of the compression force. The factor disturbing the measurement of the compressive force is the friction occurring on the faces of the cylindrical sample.

Under ideal conditions, during a static compression test, the diameter of a cylindrical specimen would change uniformly over its entire height. However, for real conditions, the effect of friction causes the sample material to flow unevenly (a barrel is formed), which is a result of blocking the flow of material on the front contact surfaces of the sample with the substrate and the punch. The static tensile test does not have this disadvantage, however, due to the nature of the material, it is impossible to carry out.

**Figure 12:**
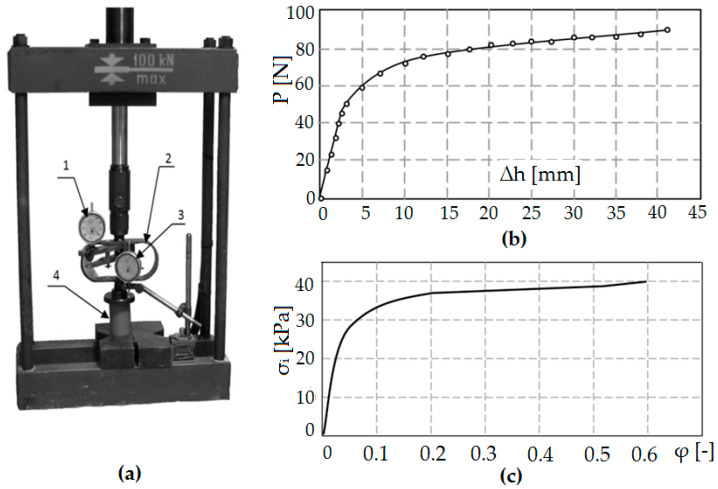
Stand for static compression test on the model material: 1—bow dynamometer, 2—dial displacement sensor, 3—compressed sample, and 4—dial gauge calibrated in force units (**a**), change of the compressive force *P* as a function of the height change Δ*h* of the cylindrical sample (**b**), the actual compression diagram for the model material (**c**).

The compression test was carried out on the stand shown in Figure 12a. A cylindrical sample with the initial height *h*_0_ = 50 mm and the initial diameter *d*_0_ = 2*r*_0_ = 40 mm was used for the tests. On the basis of the compression test, the dependence of the force as a function of the change of sample height *P* = *f* (Δ*h*) was developed (Figure 12b). Using the results of the compression test and appropriate dependencies from the works of Dawidenkow and Spiridonowa [39,43,46], a material model was developed in the form of an actual compression diagram (Figure 12c). The obtained material characteristics for the model material are similar to the material characteristics of C 45 steel.

The aim of the model research was to determine the relationship between the vertical angle of triangular asperities and the elastic recovery of the model material. The burnishing element in the test was a flat punch mounted on a hydraulic press. The tests allowed for visual assessment of material displacement, numerical determination of the size of deformation, and elastic recovery of the material. It is also possible to determine the magnitude of stresses and the direction of material flow. The samples made of model material (plasticine) were characterized by the vertical angles of asperities from *θ* = 60° to *θ* = 150°, every 15°. Each sample had three asperities with the same vertical angles and was cut along the longer sides to apply a coordination grid. Photos of samples before, during, and after the burnishing process are presented in Table 2.

Then, the samples were placed in the matrix, which task was to properly position the sample in relation to the punch and to ensure appropriate boundary and initial conditions. The stamp was displaced, causing the deformation of the vertices of the triangular asperities and the deformation of the coordination grid. The sample and stamp were covered with a layer of talcum powder to reduce friction between them. Using a coordination grid applied to the samples, it was possible to perform appropriate measurements of the elastic recovery of the material.

**Figure 13:**
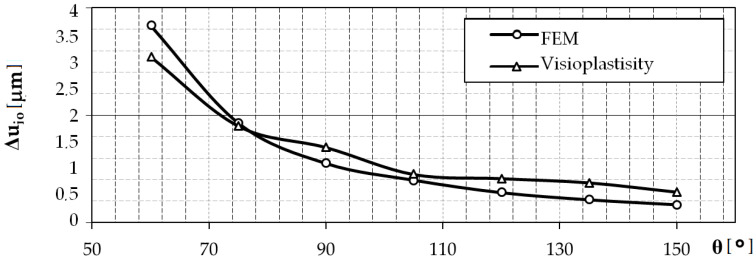
Influence of the vertical angle of surface asperities, *θ*, on the material elastic recovery Δ*u_i_*_0_.

The obtained results are presented in the form of a graph in Figure 13. The model tests of the elastic recovery of the model material using the visioplasticity method show that with the increase of the value of the vertical angle of the surface asperities, the value of the elastic recovery of the material decreases. The results were confirmed by numerical analysis. In some areas, the results are very close. The differences may be due to simplifications and assumptions made during creating a computer model. The accuracy of FEM calculations is influenced, among others, by the ratio of the side length of the finite elements used to discretize the object (object and tool), mesh refinement (mainly in areas of strong geometric or physical nonlinearity), and the finite element shape function. Although, in order to determine the significance of the influence of these factors and to determine an effective discrete model for the case of roller burnishing of a rough surface, sensitivity analysis was performed. As a result, acceptable gaps between the results were obtained.

### 4.2. Computer Simulation of the Phenomenon of Elastic Recovery Depending on the Vertical Angle of the Asperities

**Figure 14:**
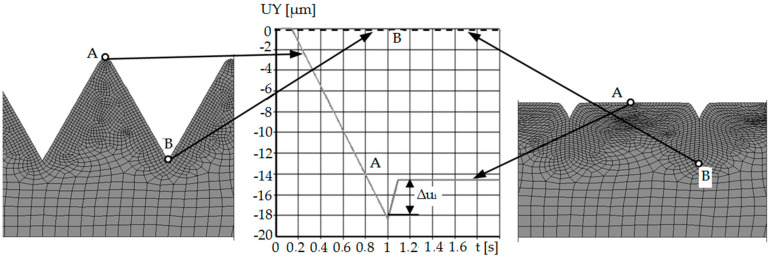
Diagram of displacement along the *Y* axis of selected nodes of asperity with a vertical angle in the process of crushing with a flat punch: A—top of asperity, B—bottom between asperities.

Computer simulations were developed for the same values of the asperities’ peak angles as in the research on the model material. The calculations were made in two stages. In the first stage, triangular irregularities were burnished, and in the second stage, the burnishing tool was moved away. It was burnished to a depth of *a* = 0.5 h, where h is the height of the triangular asperity. This approach to the problem made it possible to calculate the value of the material elastic recovery by determining the displacement of the nodal point of the peaks of the asperity. The difference between the node displacement modules at the moment of contact with the burnishing tool and after the tool is fully retracted is the desired result (Figure 14). Table 3 presents selected results from 2D computer simulations before, during, and after the burnishing process, showing displacement maps.

**Figure 15:**
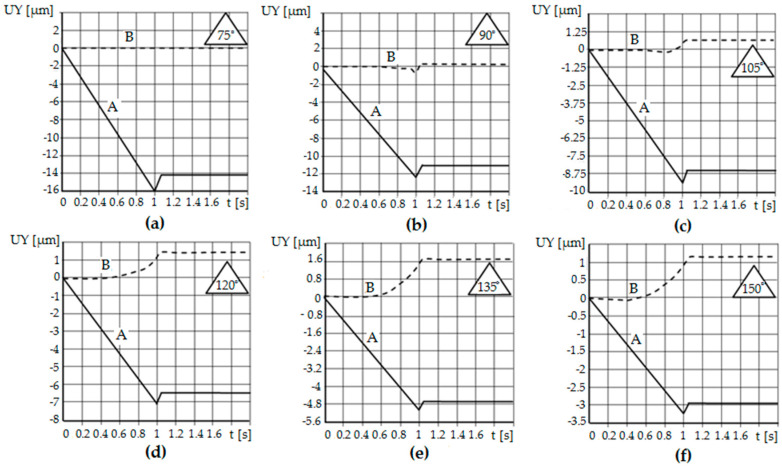
Diagram of displacement along the Y axis of selected asperity nodes with the vertical angle *θ* = 75° (**a**), *θ* = 90° (**b**), *θ* = 105° (**c**), *θ* = 120° (**d**), *θ* = 135° (**e**), *θ* = 150° (**f**), crushed with a flat punch: A—peak of asperity, B—bottom between asperities.

Figure 14 and Figure 15 show diagrams of the displacement of the node lying on the contact surface (the highest point of asperity) along the *Y* axis.

In the initial phase of the process, the node was slowly displaced due to the impact of the tool on the burnished material. The asperity was deforming. At the moment when the burnishing tool shifted to half of the roughness, the observed node reached the highest value of displacement. The first stage of numerical analysis has been completed. In the second step, when the time exceeded 1 s, the crushing tool was moved back.

The phenomenon of elastic recovery of the material is visible and then the stabilization of the position of the node (A) (the peak of the roughness), which has stopped moving along the *Y* axis. Similar results were obtained for the cases of crushing the remaining asperities with different vertical angles. When analyzing the node located between the asperities (B) (on the bottom), no elastic recovery was found for the case of asperities with a constant vertical angle and height.

The difference between the individual simulation results concerns the displacement value, which is related to the burnishing depth. The burnishing depth resulted from the geometry of the outline, i.e., the vertical angle of the asperities, and thus its height. The distance of the asperity was the same in each case. The elastic recovery also changed its value—the greater the vertical angle of the roughness, the smaller the value of the elastic recovery.

Computer simulations made it possible to observe and quantify the amount of material elastic recovery during the burnishing process. It is possible to check the results at any time during the process. It was confirmed that with the increase of the value of the vertical angle of the surface roughness, the value of the elastic recovery of the material is smaller. Moreover, using the developed numerical model, it is possible to qualitatively and quantitatively determine how much the bottoms between surface asperities rise in the process of their crushing. In cases where the vertical angles of the asperities were equal to *θ* = 60° and *θ* = 75°, the indentations of the asperities (bottom) did not rise, and the material core remained undeformed, and with complete deformation, deformed asperities are visible, separated from each other by gaps (planes of discontinuity) at depth *a* = 0.5 *R_t_*. In the range of vertical angle *θ* = 90–135°, the plastic deformation zone increased to encompass the material core. The indentations between the asperities (bottom) rise (Figure 15), and with complete deformation, gaps are visible, but of less depth. For the angle *θ* = 150°, deformations of the roughness and the core of the material occur, and the cavities between the asperities (bottoms) rise completely.

## 5. Conclusions

The phenomenon of elastic recovery of the material during burnishing is a very complicated issue. The presented results of model tests, simulations, and analyses do not reflect the complexity of the problem. They confirm that this phenomenon occurs and there are methods by which it is possible to try to quantify the dependence of the elastic recovery on, for example, the apex angle of the inequality or deviations of the inequality outline. In the conducted analyses, only the elastic recovery of the burnished material was taken into account. The spring-return occurring in the tool material was omitted, treating the tool as perfectly rigid. It should also be noted that the phenomenon of elastic recovery probably also takes place during the treatments preceding burnishing. It is not possible to continuously measure the elastic recovery during modeling or experimental studies. This possibility is provided by computer simulations using the finite element method. However, the history of the material was not taken into account in the simulations performed. Model tests and computer simulations are a cheaper way than carrying out experimental tests each time. They give a guideline to consider the material elastic recovery when designing the burnishing process, especially in the case of fine machining. Then, when calculating the burnishing depth, its value should be increased by the elastic recovery of the material. Based on the research, the following conclusions can be drawn:

- Knowledge about influence of determined profile parameters on the physical phenomena that occur in the burnishing process is unknown. It is important because they impact on the quality of the formed product’s surface layer, tribological properties, and fatigue issues. Many physical phenomena whose observation or measurement is very difficult or impossible take place during processes of burnishing, such as the pressure in contact zones, the friction forces, strain and stresses, elastic recovery, the slip and contact zones, and states of displacement.
- After passing the burnishing tool, visco-plastic deformations remain in the workpiece, while the elastic deformations change and settle at the level corresponding to the new equilibrium state of the object. Thus, the states of displacements, stresses, and strains in the tested object during and after the end of the process differ significantly, because after the process is completed, the nodes are displaced in the elastic range. In the case of higher asperities, the displacement is greater than in the case of those with a height deviation. The opposite is the case for a distance deviation. These issues are very complex and require further analysis.
- The obtained regression equations of the dependence of the material elastic recovery after burnishing (Δ*u*_0_) from the deviations of height (Δ*h_t_*) and distance (Δ*s_t_*) of asperities after turning are reasonable for specific angles of asperities (*θ* = 60°) for C45 steel.
- The increase of the deviation of the asperities’ height causes a decrease in the value of elastic recovery, while the opposite situation occurs when the deviation of the distance was analyzed.
- Comparing the values of elastic recovery for an asperity with constant geometry, *A1*, and changeable geometry, *A2*, it can be seen that the value of elastic recovery was higher for asperity *A1*.
- It was confirmed that with the increase of the value of the vertical angle of the surface roughness, the value of the elastic recovery of the material is smaller. Moreover, using the developed numerical model, it is possible to qualitatively and quantitatively determine how much the bottoms between surface asperities rise in the process of their crushing.

## Figures and Tables

**Table 1:** Experimental plan with real and coded values.

	1	2	3	4	5	6	7	8	9	10	11	12	13
Δ*h_t_*	0.01	0.01	0.03	0.03	0.02	0.02	0.04	0	0.02	0.02	0.02	0.02	0.02
Δ*s_t_*	0.05	0.15	0.05	0.15	0.2	0	0.1	0.1	0.1	0.1	0.1	0.1	0.1
Δ*h_t_*	−1	−1	+1	+1	0	0	+1.414	−1.414	0	0	0	0	0
Δ*s_t_*	−1	+1	−1	+1	+1.414	−1.414	0	0	0	0	0	0	0

**Table 2:** View of the exemplary samples.

	Before Burnishing	During Process	After Burnishing
*θ* = 60°	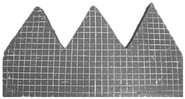	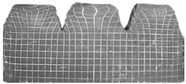	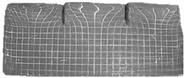
*θ* = 150°	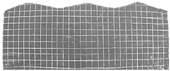	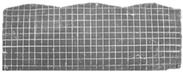	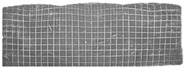

**Table 3:** Results of sample computer simulations.

	Before Burnishing	During Process	After Burnishing
*θ* = 60°	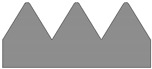	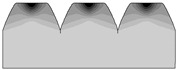	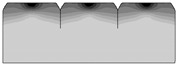
*θ* = 150°	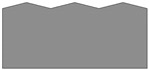	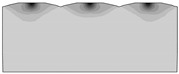	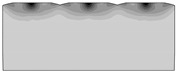

## Nomenclature

Δ*ϕ^(E)^*	increment of logarithmic strain in elastic stage
*ϕ^(E)^*	logarithmic strain in elastic stage
Δ*u_i_*	elastic recovery
*θ*	vertical angle
*σ_p_*	yield stress
